# Biomimetic Polymer Surfaces by High Resolution Molding of the Wings of Different Cicadas

**DOI:** 10.3390/ma14081910

**Published:** 2021-04-11

**Authors:** Graham Reid, James C. McCormack, Olivier Habimana, Fabian Bayer, Catherine Goromonzi, Eoin Casey, Aidan Cowley, Susan M. Kelleher

**Affiliations:** 1 School of Chemistry, University College Dublin, Dublin 4, Ireland; graham.reid@ucdconnect.ie (G.R.); james.mc-cormack@ucdconnect.ie (J.C.M.); 2 School of Biological Sciences, The University of Hong Kong, Pokfulam Road, Hong Kong, China; ohabim@hku.hk; 3 School of Electronic Engineering, Dublin City University, Glasnevin, Dublin 9, Ireland; fabian.t.bayer@googlemail.com (F.B.); CatherineGoromonzi@hotmail.com (C.G.); aidan.cowley@esa.int (A.C.); 4 School of Chemical and Bioprocess Engineering, University College Dublin, Dublin 4, Ireland; eoin.casey@ucd.ie; 5 School of Chemical Sciences, Dublin City University, Glasnevin, Dublin 9, Ireland

**Keywords:** replica molding, cicada wings, biomimetic, microstructured surfaces

## Abstract

Recent studies have shown that insect wings have evolved to have micro- and nanoscale structures on the wing surface, and biomimetic research aims to transfer such structures to application-specific materials. Herein, we describe a simple and cost-effective method of replica molding the wing topographies of four cicada species using UV-curable polymers. Different polymer blends of polyethylene glycol diacrylate and polypropylene glycol diacrylate were used as molding materials and a molding chamber was designed to precisely control the x, y, and z dimensions. Analysis by scanning electron microscopy showed that structures ranged from 148 to 854 nm in diameter, with a height range of 191–2368 nm, and wing patterns were transferred with high fidelity to the crosslinked polymer. Finally, bacterial cell studies show that the wing replicas possess the same antibacterial effect as the cicada wing from which they were molded. Overall, this work shows a quick and simple method for patterning UV-curable polymers without the use of expensive equipment, making it a highly accessible means of producing microstructured materials with biological properties.

## 1. Introduction

In recent years, much attention has been given to looking towards the natural world for inspiration for solutions to many scientific problems. Researchers have begun investigating the surface features of wings of insects such as cicadas, dragonflies, and butterflies, and have identified complex micro- and nanoscale topography which provide the surfaces with unique optical, biological and physical properties [1,2,3]. Direct investigation of the wings has shown that the topographical features are responsible for properties such as the hydrophobicity, antibacterial activity and anti-reflectivity of the wings [4,5]. The use of such wings to influence bacterial cell death is a fascinating and important field of research, as the proposed theory, whereby the physical interaction of the surface features with the bacteria is the reason for ultimate cell damage/death. This means that controlled fabrication of surfaces with features which result in an antibacterial effect could ultimately have the potential to target bacteria which have developed resistance to chemical antibacterial interventions. For the most part, the topographical features, which are present on natural insect wings that are shown to possess antibacterial activity, are arrays of pillar-like structures which vary in height, diameter and spacing depending on the species. Ivanova et al. were one of the first teams to report on the phenomenon and stated that individual *Pseudomonas aeruginosa* cells were killed by the surface within approximately three minutes of contact with the wing of *Psaltoda claripennis* [6]. Additional work by their team reported on the activity of other similarly structured surfaces, such as black silicon and gecko hairs [7].

Their work opened the door to many further studies on the interaction of bacteria with the wings of insects. In 2015, Nowlin et al. studied the interaction between *Saccharomyces cerevisiae* on cicada and dragonfly wings [8]. They discovered that the features on the surface of these wings engaged with the yeast in a manner which resulted in the yeast cell rupturing. Our own work, published in 2016, studied three species of cicada, *Megapomponia intermedia*, *Ayuthia spectabile* and *Cryptotympana aquila*, and found a correlation between the size of the pillars present on the wings and the antibacterial activity, with smaller features providing the greatest effect [4].

In 2017, Bandara et al. carried out detailed analysis of the interaction of *Escherichia coli* with the surface features present on the wing of the *Orthetrum villosovittatum* dragonfly, in turn shedding new light on the possible mode of action of the features on cell damage/death, with their experiments [9]. Following this, in their 2019 paper, Shahali et al. studied the antibacterial activity of the wings of three different cicadas, *Psaltoda claripennis*, *Aleeta curvicosta*, and *Palapsalta eyrei,* in addition to biomimetic titanium nanopillars [10]. They reported that overall, a higher bacterial killing efficacy was observed on the wings in shorter time durations (sub 4 h for both *Staphylococcus aureus* and *Pseudomonas aeruginosa* cells). No quantitative cell death testing was carried out on the titanium structures. More recently still, further work by Bandara et al. investigated the interface between the features present on unspecified dragonfly wings and *E. coli* cells using high resolution helium ion microscopy [11]. This work highlighted the close connectivity between the pillars on the surface and the cell membrane. However, the authors refrained from drawing additional conclusions on the role this interaction played on the cell fate. 

To date, there has been significant work carried out looking at the effect of nano- and microscale patterning of surfaces on bacterial cell adhesion, with a clear demonstration between the presence of surface topography and a reduction in bacterial cell adhesion [12,13,14,15,16]. However, there still remains a need to fully understand the role these topography dimensions play on bacterial cell fate upon adhesion, both in a general sense but also in a targeted manner. This is particularly important as many researchers have reported differences between the level of fatal interaction of Gram-negative and Gram-positive cells. Theoretical work looking at the interaction between the cell membrane and nanoscale pillars has been carried out. Xue et al. completed computational work which proposed that periodic nanoscale surface protrusions would be sufficient to prevent or reduce bacterial cell adhesion, while also reporting that full cell adhesion would not be possible [17]. Pogodin et al. hypothesized that the stretching and, therefore, ultimate rupturing of the bacterial cell membrane occurred when there was sufficient distance between the pillars [18]. However, this does not agree with our own experimental findings where pillars that were close together did more damage to bacteria. Similarly, recent work by Heckmann et al. showed that closely packed microscale pillars of polydimethylsiloxane (PDMS) also caused greater cell damage against *E. coli* and *S. aureus* than pillars spaced further apart [19]. The work by Bandara in 2017 proposes that the membrane stress is a result of a combination of strong adhesion between pillars and the bacterium extracellular membrane as well as shear force when immobilized bacteria attempt to move across the structure, and not, in fact, as a result of direct contact of the bacterial cell membrane with the nanopillars [9]. The effect of surface chemistry, in addition to topographic effects, cannot be overlooked when discussing bacterial adhesion on structured materials. Roman-Kustas et al. have completed some important work on looking at both the chemical composition of cicada wings, as well as how changes in surface chemistry affects bacterial cell adhesion [20,21]. Their work concluded that the surface chemical constituents on the wing play a role in the antimicrobial activity of the wings, in addition to the surface structures.

It is clear that further work is needed to understand the role topography plays on the antimicrobial activity of nano- and microstructured surfaces to garner a full appreciation of the mechanisms involved, and how best to tune materials to be most effective. The need for biomimetic materials, which can be adapted to alter the surface chemistry and topographical dimensions, is essential. Methods such as replica molding or etching allow functional surface topography to be transferred to relevant surfaces where their beneficial properties can be studied [22,23,24,25]. The molding of surfaces that are covered in nano- and micron-scale features has already been achieved using various techniques such as soft lithography, photolithography, injection molding, hot embossing, atomic layer deposition, capillary force lithography and ultraviolet nanoimprint lithography (NIL) [26,27,28,29,30,31,32,33,34,35]. Depending on the molding polymer and technique employed, feature resolutions of 100 nm can be achieved, with reports of features as small as 6 nm in diameter [36,37,38]. Zhang et al. used wings as templates for nanoimprint lithography to make a negative mold in polymethyl methacrylate (PMMA), followed by a deposition of a thick gold layer, to produce a gold replica of the wing itself [39]. Similarly, Wang et al. reported on a rapid fabrication method for producing a PMMA replica of a cicada wing and studied its anti-reflection properties. Hong et al. carried out a combination of hot embossing and UV nanoimprinting to produce a polymeric replica of a cicada wing in a UV-curable resin, using a 6-stage process [40]. More recently, Xie et al. have produced a polystyrene replica of a cicada wing using a sequence of molding which included nickel-plating and melt injecting to construct a sample which possessed a hexagonally packed array of micron-scale pillars, but lacked the fidelity of the tip radius present on the cicada wing [41].

There are advantages and disadvantages to the above reported techniques, but the main drawbacks are related to the time consumed due to increased steps in the procedure and overall fabrication costs. This is especially true if expensive instrumentation is required to create molding templates using ion-based lithography methods, high pressures required for NIL, or resists for photolithography [37,42,43,44,45]. While the long-term development of these types of surfaces will rely on scalable processes, and not molding of natural materials, we saw the need to better understand the resolution of molding processes on various structures, as it was clear that the role topography played, and the development of surfaces with varied topographies, would be an important next step in this field. We utilized UV-curable polymers, as this curing process was fast and cost-effective, and required simple equipment to achieve high-fidelity replicas. Previous work using UV-curable polymers in the context of fabricating replicas of surfaces included replicating lotus leaves, carbon nanotubes, silicon, glass and aluminum [46,47,48,49,50,51,52,53]. UV-curable polymers have additional benefits of not requiring heating or pressure when being molded, which could be important to avoid in a situation where the natural sample is delicate or not readily available. Additionally, UV-curable materials are highly tunable in terms of their physical and chemical properties (swelling, hydrophobicity, degradation patterns). We developed a simple two-step process to fabricate molds of five different cicada wing topographies. We identified limitations in drop cast molding of tightly packed features and developed a molding method to overcome this by using low-viscosity polymers and a molding chamber to control the molding process. Being able to produce surfaces where sub-200 nm diameter features can be replicated is important to enable these materials to be tested for activity, as smaller features have thus far been reported as some of the most active against bacteria [4,19]. Initial biological testing on a replica sample shows that the polymeric molds possess the same antibacterial activity as the corresponding wing. Our process would be available to many research groups as a tool to make biomimetic surfaces in tunable polymeric materials. Additionally, the process we have developed also gives long-range resolution of features and the initial negative mold of the wing can be reused to generate numerous polymer replicas.

## 2. Materials and Methods

### 2.1. Materials

Acetone (99.0% anhydrous), iso-propanol (IPA) (99% anhydrous), PDMS (Sylgard 184), 2-hydroxy-4′-(2-hydroxyethoxy)-2-methylpropiophenone (98%) photoinitiator (PI), polyethylene glycol diacrylate Mw 575 gmol^−1^ (PEGDA), polypropylene glycol diacrylate Mw 800 gmol^−1^ (PPGDA), gentamicin, King B agar, and King B broths and phosphate-buffered saline (PBS) were purchased from Sigma Aldrich, Wicklow, Ireland. SYTOX Green, propidium iodide, and Syto-9 were purchased from Invitrogen, Dublin, Ireland. Polytetrafluoroethylene (PTFE) 6-well plates were purchased from Nunc, Roskilde, Denmark. Dead, dried, unmounted cicadas were purchased from insectartonline.com (Accessed 1 September 2018), and were collected by suppliers after a natural death following surfacing. The molding chamber was custom-built to our specifications by Steger GmbH in Waiblingen, Germany. The full details of the chamber design can be found in Supplementary Materials Figure S1.

### 2.2. Methods

#### 2.2.1. Preparation of Wings

Small sections (approx. 2 × 2 cm) of the wings were cut from the insect, cleaned by sonicating in deionized water for 20 min, and then gently dried under a stream of nitrogen. The wings were then stored under vacuum until used.

#### 2.2.2. Preparation of Polymer Precursor Solution

To prepare the polymer precursor solution, 1% (*w*/*v*) 2-hydroxy-4′-(2-hydroxyethoxy)-2-methylpropiophenone PI was placed in a glass vial and dissolved in 2–3 drops of acetone. To this solution, either PPGDA or PEGDA (both liquids at room temperature) (1 mL) was added and vortexed (for 1 min) to ensure a homogenous solution was generated. For blends, the required amount of PPGDA/PEGDA was added by volume. The vial was put into a desiccator and a vacuum pulled for 30 min to degas the solution and remove the acetone.

#### 2.2.3. Drop-Cast Method for Replica Molding

For drop-cast molding, cleaned wings were immobilized on a microscope slide with a silicone adhesive. Prepolymer solution (approx. 200 µL) was drop-cast onto the top surface of the wing. It was essential that the surface tension of the drop cast polymer was not broken, otherwise the polymer solution would travel under the wing and the primary mold would be too thin to easily handle. This sample was then transferred to a nitrogen glovebox and cured under UV light (365 nm) for 30 min for PEGDA solutions and 60 min for PPGDA or blend solutions at 8 cm from the light source. After curing, the primary mold was gently separated from the wing using sharp tweezers and a scalpel. The primary mold was then inverted (patterned side facing up) and immobilized on a microscope slide. Prepolymer solution (20–100 µL) was drop-cast onto the primary mold (depending on the desired size of the secondary mold). The secondary mold was cured and separated using the procedure above.

#### 2.2.4. Molding in the Molding Chamber

**Scheme 1:**
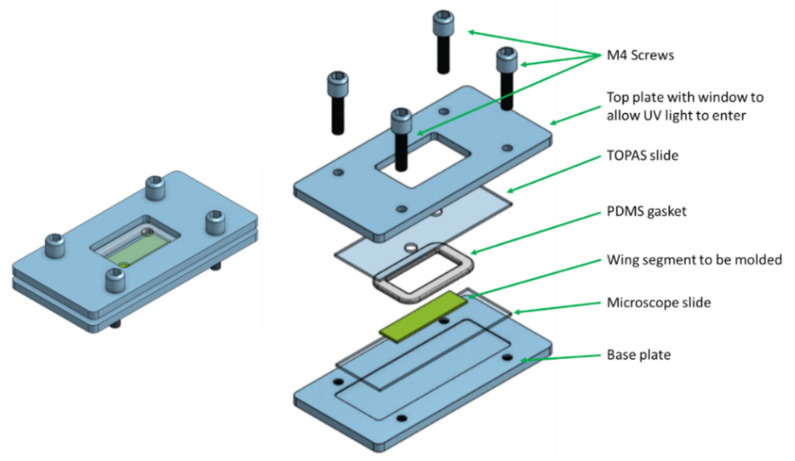
Schematic of the replica molding chamber.

Scheme 1 shows the molding chamber set up. PDMS and elastomer curing agent solution was mixed at a ratio of 10:1, the solution was degassed to remove bubbles by placing the vial into a desiccator and pulling a vacuum for 30 min. This mixture was then cured using a PMMA guide to form 2 mm thick gaskets (which controlled the z dimension of the resulting mold). The PDMS was then cut to the desired x and y dimensions using a scalpel. The wing, or primary mold, was immobilized on a glass slide that was roughened to aid adhesion. The PDMS gasket was placed over the template and a 2 mm thick TOPAS slide was placed over the PDMS in line with the underlying glass slide. Before aligning, two holes were punched in the TOPAS slide at corners diagonally from each other to allow pipetting of the prepolymer in and air out. Both slides were secured in the molding chamber by the top and bottom steel plates using four hex head bolts. Once moved to a nitrogen atmosphere, prepolymer was pipetted over the template and care was taken to avoid bubbles. Polymerization was then carried out under UV light in the same manner as described for drop cast molding.

#### 2.2.5. Water Contact Angle and Surface Free Energy Measurements

Water contact angles of cicada wing surfaces, primary molds and secondary molds were measured using the sessile drop method. The measurements were carried out using an FTA200 Dynamic Contact Angle Analyser. Wing and mold samples were immobilized and trimmed with a scalpel if they obscured the camera of the analyzer. Water droplets (1–2 μL) were used to obtain measurements and an average of 3 measurements were taken from three different sections of the sample.

For surface free energy (SFE) and contact angle measurements of planar PEG and PPG samples, a Krüss DSA 025 Drop Shape Analyser was used. Four planar molds of each polymer were analyzed using deionized water and diiodomethane as the test liquids for SFE measurements. Samples were UV-cured, rinsed with acetone, and then stored in a desiccator for 16 h. Nine drops (2 µL) of each test liquid were used on each sample to obtain an SFE value using the OWRK method.

#### 2.2.6. Scanning Electron Microscopy

Both a Karl Zeiss Ultra field emission scanning electron microscope and a Karl Zeiss EVO series scanning electron microscope (using the secondary electron detector at an accelerating voltage of 5–10 kV) were used to image the wings and polymer mold topographies. Tilt images were acquired with tilt correction activated at 45°. For all scanning electron microscopy (SEM) imaging, all samples were coated with approximately 15 nm of gold using either a sputter coater Scancoat Six instrument (HHV Scancoat, Crawley, UK) or an Electron Microscopy Sciences K550 instrument (Hatfield, PA, USA).

#### 2.2.7. Image Analysis

All SEM images were analyzed using ImageJ (https://imagej.nih.gov/ij/, accessed on 2 January 2019). Top-down images were taken and used to determine the diameter of both pillars (on wings and secondary molds) and pores (on primary molds). The elliptical tool in ImageJ was used to select features that were at 90° to the beam, and the Feret diameter was then measured. A minimum of 50 features were measured and an average and standard deviation were calculated. Only features where a clear distinction between adjacent features could be seen were selected for diameter analysis. For the height of pillars/features, tilted images were analyzed and the line function in ImageJ was used to measure the height of a minimum of 50 features. Only independently standing features, where the base of the feature could be seen, were selected for height analysis.

#### 2.2.8. Cell Preparation for Adhesion Assay

One Gram-negative *Pseudomonas fluorescens* PLC1701 model strain (provided by Dr Ellen L. Lagendijk, Institute of Biology, Leiden, The Netherland) and one Gram-positive *Staphylococcus epidermidis* ATCC 12228 model strain (a referenced organism from the American Type Culture Collection) were selected for bacterial adhesion assays in this study. An mCherry-expressing *P. fluorescens* was stored at −80 °C in King B broth supplemented with 20% glycerol. Independent *P. fluorescens* cultures were obtained by inoculating 100 mL King B broth supplemented with gentamicin at a final concentration of 10 µg·mL^−1^, using a single colony of a previously grown culture on King B agar at 28 °C. Independent *S. epidermidis* cultures were obtained by inoculating 100 mL King B broth using a single colony of a previously grown culture on King B agar at 28 °C. Both inoculated mediums were then incubated at 30 °C with shaking at 75 rpm for 16 h until the cell culture reached an optical density (OD_600nm_) between 0.8 and 1.2.

#### 2.2.9. Static Bioadhesion Assays

Cell concentrations were standardized for each strain and adhesion experiment by first centrifuging *P. fluorescens* and *S. epidermidis* overnight cultures at 5000 rpm for 10 min using a Hettich Universal 320R centrifuge, and by re-suspending cell pellets in sterile PBS. Cell suspensions were then diluted using PBS to an OD600 of 0.2, which corresponds to an inoculum of approximately 10^8^ cells mL^−1^. Static adhesion assays were performed on samples of the ME cicada wing and PEG replicas of the ME wing. PEG samples were allowed to swell overnight in PBS prior to being used in the cell adhesion studies. This ensured that there was no swelling of the surface structures when testing was underway. Samples were cut into small sections and immobilized at the bottom of PTFE 6-well plates. Bacterial adhesion was initiated by adding 4 mL of freshly prepared cell suspensions of *P. fluorescens* or *S. epidermidis* cells in individual wells. Wells were then left to rest for 30 min at room temperature. To end the adhesion experiments, 4 mL sterile PBS solution was added to individual wells, followed by a systematic removal of a 4 mL volume of diluted bacterial suspension. This process was repeated three times for each well of the 6-well plate. Static adhesion was performed three times using independent *P. fluorescens* or *S. epidermidis* cultures. Bioadhesion on each support was performed in three independent experiments, following the acquisition of at least *n* = 10 micrographs per experiment for each tested surface.

#### 2.2.10. Viability Analysis and Epi-Florescence Microscopy Analysis

To assess the degree of cell structural damage on adhered cells following bioadhesion assays, a volume of 1 μL of SYTOX Green (5 mM) was added to individual wells of the 6-well plates containing *P. fluorescens* cells. Damaged *S. epidermidis* cells were stained by adding 1 μL propidium iodide (20 mM). For visualizing total adhered *S. epidermidis* cells, 1 μL DNA-based Syto-9 stain (5 mM) was introduced to relevant wells. Stained wells were subsequently incubated at ambient temperature for 10 min in the dark prior to epi-fluorescence microscopy (Olympus BX51, Southend-on-Sea, UK) using a 10× objective. Two images were acquired for every chosen observation field using U-MNG and U-MWB filter cubes for differentiating between fluorescent mCherry-tagged and SYTOX Green-stained *Pseudomonas* cells. In the case of *Staphylococcus* cells, the U-282MNG and U-MWB filter cubes were utilized to visualize propidium iodide-positive and Syto-9-positive cells, respectively. Ten different fields of view were obtained at random points from each tested sample. Cell surface coverage (%) for mCherry-tagged, SYTOX Green, propidium iodide, and Syto-9-stained cells was determined for each tested sample using ImageJ^®^, a Java-based image processing program (1.52v). Acquired images were subsequently grayscaled and thresholded using the Multi-Thresholding macro (https://imagej.nih.gov/ij/plugins/multi-thresholder.html, accessed on 3 March 2019) which allowed each acquired image to go through 15 different thresholding methods. One thresholding method was then selected based on edge accuracy, delimitating cells from background. Cell surface coverage (%) was then determined as the percentage of solid surface covered by bacteria, based on the number of black and white pixels of thresholded images. The level of cell fitness as a consequence of their interaction with tested structured surfaces in terms of cell dead/live (D/L Ratio) was then calculated by dividing surface coverage data from SYTOX Green-positive and propidium iodide-positive with mCherry-tagged cells and Syto-9-positive cells, respectively. The data were visualized using Analyse-it^®^ (Analyse-it, Leeds, UK).

#### 2.2.11. Statistical Analysis

The statistical significance of the effect of the microstructured surface (ME wing and PEG replica) on the *P. fluorescens* or *S. epidermidis* cell damage ratio following static bioadhesion assays was assessed by means of one-way analysis of variance (ANOVA) tests with Tukey–Kramer all pair multiple comparison using Analyse-it^®^ (Analyse-it, Leeds, UK). All ANOVAs were carried out at a 5% significance level.

## 3. Results and Discussion

### 3.1. Analysis of Wings

**Figure 1:**
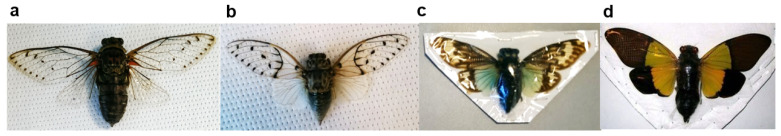
Photos of the cicada wings used in this work: (**a**) *Megapomponia intermedia* (ME); (**b**) *Ayuthia spectabile* (AY); (**c**) *Tosena splendida blue* (TO); (**d**) *Trengganua sibylla* (TR).

Four cicada species were studied in this work: *Megapomponia intermedia* (ME), *Ayuthia spectabile* (AY), *Tosena splendida blue* (TO) and *Trengganua sibylla* (TR) (Figure 1). The samples used in this paper were taken from different parts of these four cicadas. ME has completely transparent forewings and hindwings. Previous work from our team showed that the pillar structure present across these wings is identical between the forewings and hindwings, as well as from the dorsal and ventral sides [4]. The AY sample used was the transparent forewings.

**Figure 2:**
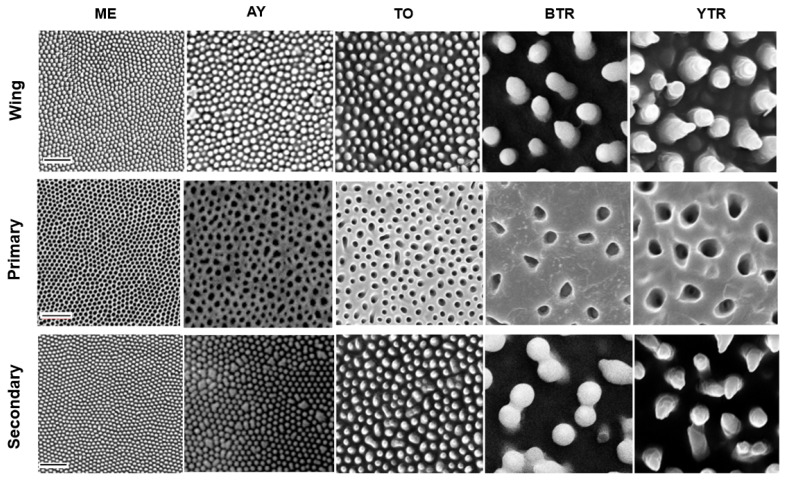
SEM images of the wings, primary molds, and secondary molds (scale bar is 1 μm and all images are the same size).

The AY sample was previously analyzed by our group alongside the ME wing. TR has both brown and yellow sections on the forewings and hindwings. Samples of both the brown (BTR) and yellow (YTR) areas were studied. Finally, the TO wing has a brown/speckled forewing and a primarily green hindwing. In this work, the green hindwing was used. Analysis of five samples (ME, AY, TO, BTR, and YTR) was carried out using SEM. All wing samples were seen to have an array of pillars on the surface in the range of 148–854 nm in diameter. ME had the smallest pillars, with a pillar diameter of 148 ± 9 nm and a pillar height of 215 ± 19 nm. AY had a pillar diameter of 209 ± 20 nm. The largest features were those found on the TR sample, with pillar heights over 2000 nm on both the YTR and BTR sections. The average pillar diameter and height for all wing samples are presented in Table 1. Tilted SEM images of the ME, AY and TO pillars revealed a conical shape with curved spherical tops (Supplementary Materials Figure S2). The pillars on BTR and YTR are an order of magnitude larger than the other species, and maintain a level of uniformity, although not as well ordered as ME. Figure 2 shows top-down SEM images of the wing samples.

The aspect ratio of the pillars gives an indication of the overall shape of the features, with all features being taller than they are wide, with the exception of the AY wing features. All wings were hydrophobic as a result of surface chemistry and the features present on the surface, with water contact angles (WCA) ranging from 103 to 117° (Table 1). These are in line with the values reported by Sun et al., who studied the wettability of 15 cicada species [5].

### 3.2. Molding Using PEGDA

**Scheme 2:**
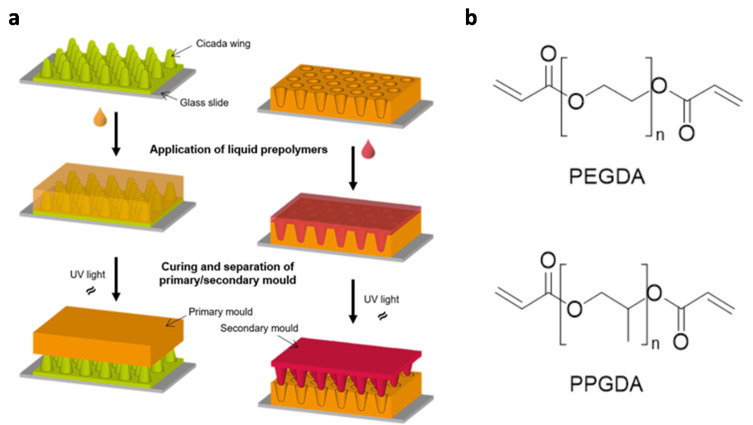
(**a**) Schematic of the replica molding process with (**b**) the chemical structures of liquid prepolymers (PEGDA and PPGDA) used for this work.

Initially, molding of all samples was performed using UV-curable, short chain PEGDA. To acquire a replica mold which had the same features as the wing, a double replica molding process was required (see Scheme 2). Initial experiments were performed using a drop-casting method, with PEGDA being used as the material for both the primary and secondary molds. Drop-casting of PEGDA onto the surface of the wing, followed by curing, resulted in PEG primary molds being produced. For all samples, the cured polymer was removed from the wing using tweezers and analyzed using SEM. Figure 2 and Supplementary Materials Figure S3 shows the top-down SEM images of the primary molds. The primary mold constituted a porous structure, where the sizes of the pores related to the size of the features present on the wing from which it was molded. Except for the AY sample (difference of ±25%), the diameter of the porous structures on the primary molds were all within a difference of (±11–16%) compared to features on the respective cicada wings.

Table 1 details the average pore diameter for all primary molds and their associated WCA. The reduction in WCA, in comparison to their corresponding wing structures, demonstrates the change in topography as well as the change in surface chemistry. The outer epicuticle of cicada wings is mainly made up of fatty acids and long chain hydrocarbons that would contribute to the inherent hydrophobic nature of the wing nanostructures [20]. However, when the WCA of primary mold PEG surfaces was investigated, they were shown to be hydrophilic (WCA < 90°). Whether the increased hydrophilicity was exclusively because of surface chemistry changes or loss of protrusions was investigated by measuring the WCAs of secondary mold PEG surfaces is discussed later in this section. The increased hydrophilicity was worth noting as it determined the wettability of the surface for the second molding step. The depth of the pores was not determined by atomic force microscopy (AFM) in this work, as AFM in this case does not accurately depict the actual pore depth; however, snap-cleave cross-section SEM images were used as an indication as to whether the replica molding process was successful (Supplementary Materials Figure S4). The pore size as extracted from the snap-cleave imaging varied largely. Difficulties in capturing these side profiles led us to believe that use of the height of pillars present on the secondary mold is a better indicator as to whether the primary mold has indeed replicated the wing structure.

Production of the secondary molds from the PEG primary molds using the same replica molding process was attempted using PEGDA. Capillary action pulled the low viscosity prepolymer solutions into networks of cavities under appropriate conditions of surface wettability [54]. In this work, the cavities in the primary molds are filled spontaneously via gravity-assisted capillary action. The driving force for capillary action, Laplace pressure, is given in Equation (1):

(1) $$P_{L}=\;\frac{\left(2\gamma \;cos\;\theta \right)}{r}$$

where γ is the surface tension of the prepolymer, *θ* is the contact angle of the prepolymer on the primary mold and *r* is the radius of the cavity opening. When Laplace pressure is in equilibrium with atmospheric pressure, capillary filling stops. Laplace pressure is inversely proportional to cavity size and can exceed 10 times the atmospheric pressure when feature sizes fall below the sub 100 nm range [55]. This facilitates complete filling of primary molds despite the air permeability of the mold material [55]. For the TO, BTR and YTR primary molds, the molding process was successful as seen from the SEM images of the secondary molds produced (Figure 2). Supplementary Materials Figure S5 shows photos of the ideal drop-casting process. For molding from the ME and AY primary samples using PEGDA, there were significant challenges in clean separation of the secondary molds, with only tiny fragments breaking off, if separation was at all possible.

It was suspected that a high surface-to-volume ratio played a large role in the attraction/increased friction between the primary and secondary molds, with the larger surface-to-volume ratio of the ME and AY molds accounting for the increased difficulty with demolding of these samples relative to the other three samples. The factors which influence the adhesion of polymeric micropillar arrays embedded in a polymeric mold has been explained thoroughly by Shahsavan et al. [56,57]. During interfacial separation, the applied separation force must overcome the friction between the pillars and the embedding material. In doing so, local contacts are broken along the sidewalls of the pillars and the planar area at the base [56,57]. Similarly, the increased local separation events which took place at the ME and AY mold interfaces, compared to the other three wing samples, increased the local adhesion of the primary and secondary mold interface. The high surface energy (62 mN·m^−1^) and high modulus of the PEG primary also played a role in the difficulties with mold separation [58,59]. Kim et al. highlighted that the polar component of surface energy may play a critical role in the clean release of the molds while replicating polymeric nanopillar arrays [37].

Polypropylene glycol diacrylate (PPGDA) was investigated as an alternative material from which to make the primary mold of the ME wing. Longer chain length, difference in moduli, surface free energy and polar fractions of PPGDA compared to PEGDA were all anticipated to aid in mold separation [37,58,59]. In other work carried out in our group, there was a difference in Young’s moduli exhibited after AFM force mapping when comparing PPG (374 ± 41 MPa) to PEG (765 ± 73 MPa) [60]. Surface free energy (SFE) measurements on planar gels confirmed the SFE of planar PPG was lower than that of planar PEG using our method (47 ± 3 mN·m^−1^ vs. 62 ± 2 mN·m^−1^); see Supplementary Materials Table S1. WCA analysis was shown to be 66 ± 3° and 46 ± 2° for planar PPG and PEG, respectively (Supplementary Materials Table S1 and Figure S6). The theoretical adhesion between PEG/PEG and PPG/PEG planar molds was compared using the harmonic mean equation (Equation (2)) and the SFE data shown in Supplementary Materials Table S1 [61]:

(2) $$W_{12}=4(\frac{\gamma_{1}^{d}\gamma_{2}^{d}}{\gamma_{1}^{d}+\gamma_{2}^{d}}+\frac{\gamma_{1}^{p}\gamma_{2}^{p}}{\gamma_{1}^{p}\gamma_{2}^{p}})$$

*W*_12_ is the adhesion between surface 1 and surface 2, *γ^d^* is the dispersive component for surface energy, *γ^p^* is the polar component for surface energy and *γ^total^* is the total surface energy. The work of adhesion at the planar gel interface was shown to decrease from 124 mN·m^−1^ to 107 mN·m^−1^ when PPG was substituted for one of the PEG surfaces.

Molding using 100% PPGDA as the primary mold and 100% PEGDA as the secondary mold was investigated. However, PPG primary molds were too thin and caused handling problems when the secondary mold was being made. For this reason, a molding chamber (Scheme 1) was developed, within which the molding was set in a confined space, where control of the x, y and z dimensions was possible. The confinement of the prepolymer limited spreading across the wing samples, facilitating control of the overall thickness of the resulting mold. The main benefits of this chamber included a leak-proof system, an interchangeable PDMS gasket for determining the x, y and z dimensions of the mold being produced, and the ability to add prepolymer solution via an inlet when under a nitrogen environment to eliminate the presence of air bubbles.

Molding in this chamber can take place for both primary and secondary mold production, and the dimensions of the mold are controlled by the dimensions of the PDMS gasket insert used. The PPG primary molds produced using this chamber were thicker than those molded without the chamber as the presence of the gasket enabled samples with a controlled thickness to be produced. For this work, 2 mm thick PPG primary molds were used. The molding chamber was used to produce a primary mold from the ME wing, and subsequently used to make a secondary mold. For this process, there was no difficulty in removing the secondary mold from the primary mold and preparation of larger samples was possible (Supplementary Materials Figure S7). The dimensions of all the secondary molds produced from the wing samples, as well as WCAs, are reported in Table 1.

A SEM image and the diameter measurements of a secondary mold of the AY retrieved from a fragment produced from the drop-cast molding method above has been added to Table 1 to show that even in unsuccessful samples, the replication fidelity is very good (the pore size of AY primary was 156 ± 14 nm and the diameter of the AY secondary mold was 156 ± 10 nm). The height of the AY secondary fragment was unable to be obtained due to sample loss prior to further imaging. The molding of the AY wing using the chamber was not attempted in this work. Figure 2 shows the side-by-side comparison of the SEM images of the wings, the primary molds, and the secondary molds. Direct visual comparison of the SEM images for all samples indicated that the molding was highly successful, with excellent replicas of the wings made in PEG. Directly comparing the feature sizes of the pillars in Table 1 supports this conclusion. Although there were differences between the wing and the secondary mold structure diameters (ME ± 4%, AY ± 25%, TO ± 9%, BTR ± 9%, YTR ± 13%) and also with wing structure heights compared to molded (ME ± 10%, TO ± 9%, BTR ± 1%, YTR ± 13%), the small amount of shrinkage which can happen with UV-curable materials in combination with the natural variation of the wing leads us to believe these are insignificant [62,63,64]. The molding method also enabled replica molding of the larger, micron-scale features on the wings, as seen in Supplementary Materials Figure S8. In addition, where calculated, the aspect ratio remains similar to that reported for the wing, showing that there is no collapse of pillars or indeed excessive shrinkage of features. The WCAs of the secondary molds were measured (Table 1) and were shown to be generally similar to those of the primary molds, but not as high as the wing. The commonly used Wenzel model for measuring WCA on rough surfaces states that the apparent WCA will usually decrease when <90°, as the water will fill any pockets of air around the nanostructures, increasing contact between the solid and liquid interfaces [65]. While they are structured, and a higher WCA may be expected, the surface chemistry of the PEG material results in a lower WCA than those of the waxy wings [66].

We were interested in the reason behind the difficulty in separation of the ME and AY molds when the PEG was used as both the primary and the secondary mold during drop-cast molding. The major difference observed between the ME and AY samples and the other samples was the topographical dimensions; the features on the ME and AY were clearly much smaller in both diameter and height. It was therefore expected that feature size might be the reason for challenging separation of these. We proceeded to make 3D models of the pillar arrays of all samples to calculate the surface area of the protrusions and the number of pillars per micron squared on each sample (Supplementary Materials Figure S9). It was anticipated that the smaller ME and AY pillars would have greater surface-to-volume ratio, and therefore greater PEG–PEG favorable interactions, which would explain the difficulty in separation. However, as seen in Supplementary Materials Figure S10, the surface area of the larger pillars (BTR, YTR) was greater than the ME and AY molds. This led us to believe that there were additional factors beyond surface chemistry to consider.

Analysis of the density of the pillars on the surface (i.e., the number of pillars per square micron) showed that the density of the pillars across the surfaces varied. ME and AY wing samples have substantially more pillars per square micron than the other samples, with ME having 47 (46.6)/µm^2^, and AY having 19 (18.8)/µm^2^, compared to 10 (9.9)/µm^2^ for TO and 1 (0.9)/µm^2^ for both BTR and YTR (Supplementary Materials Figure S10). The density of pillars takes into account both the diameter and the spacing of the pillars; therefore, these factors seem to play a large role in the ability of the molds to separate. We hypothesize that in samples with features where the density is less than 10 pillars per square micron, that separation using the PEG–PEG cast molding method would be possible. However, where the density of features increases to over 15, the straightforward separation and production of large, unbroken samples becomes challenging.

It is clear to see that there is more than just one factor at play when it comes to the ability of molds to be separated in this replica molding work, as when the chemistry of the primary mold is changed to provide greater unfavorable interactions between the molds (changing the PEG primary mold to a PPG primary mold), separation of the ME secondary mold from the primary mold was possible. This ability of the surface chemistry to override the physical properties of the surface is commonly seen in the use of release agents in soft lithography molding work (silicon, silane, release agents). More in-depth analysis of the forces involved between the patterned surfaces and the role of surface chemistry in enabling separation of molds on this scale will be the work of future studies.

**Figure 3:**
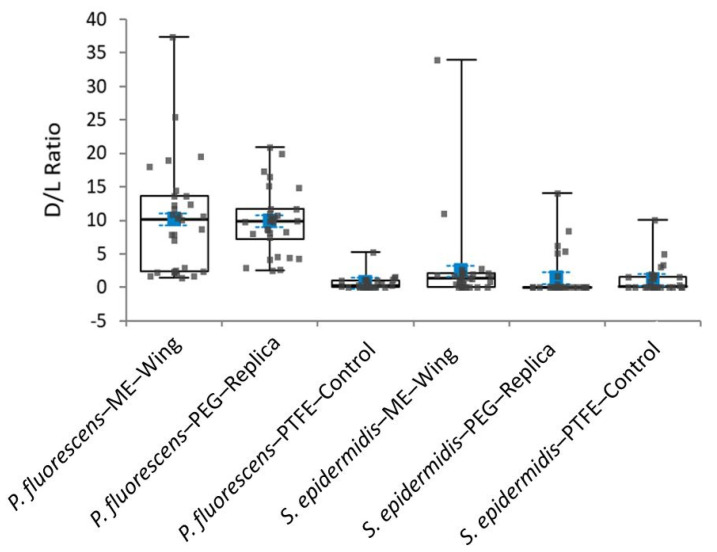
Ratio of dead/damaged cells to live cells of adhered *Pseudomonas fluorescens* and *Staphylococcus epidermidis* cells onto microstructured ME wing, PEG replica and a non-structured PTFE control surface. Cell dead/live ratios (D/L Ratio) were calculated by dividing surface coverage data from SYTOX Green-positive and propidium iodide-positive with mCherry-tagged cells and Syto-9-positive cells, respectively. Box plots show the interquartile range, black whiskers indicate standard deviation, and black lines indicate median. Internal blue dots represent the mean and the internal error bars represent standard error of the mean.

Previous work in this area showed that microstructured PDMS surfaces could reduce bacterial adherence when structures were comparable in size to the bacterial cells tested [12,19]. Along with the antibiofouling properties of micropatterned surfaces, *E. coli* has been shown to be susceptible to cell death when structures on a replicated polymer surface are smaller in diameter and spacing than the bacterial cells tested [19]. Having produced a replica mold of a number of cicada wings, we then proceeded to test the antibacterial activity of a PEG replica of a cicada wing known to possess antibacterial properties, the ME wing [6]. The cell dead/live (D/L) ratios presented in Figure 3 were calculated following static bioadhesion assays combined with strategic staining of *Pseudomonas fluorescens* and *Staphylococcus epidermidis* (model organisms for determining the level of cell fitness when interacting with the microstructured ME wing, PEG replica and PTFE control surfaces). Compared to non-structured PTFE control surfaces, the interaction of cells onto ME wing surfaces led to an increased ratio of dead/damaged cells (*p* < 0.05) for *P. fluorescens* (*p* < 0.0001); however, no such increase was observed for *S. epidermidis* cells.

Further comparison of the differences in dead/live cells ratio between *P. fluorescens* and *S. epidermidis* suggests that the ME wing may be more effective in damaging Gram-negative compared to Gram-positive cells (*p* < 0.0001). The susceptibility of Gram-negative cells towards ME wing microstructures, as observed in our previous study, may be explained by its cell wall structure, which is known to lack a thick peptidoglycan cell wall typically characterized by Gram-positive organisms [4]. The very nature of the rigid Gram-positive cell wall provides the cell with exceptional protection against mechanical stress and cell lysis with unparalleled adhesion functionality [67]. This supports the idea that Gram-negative cells are more susceptible to cell death as a result of interaction with microscale topographies, when compared to Gram-positive cells. As confirmed by our observation, no significant increase in dead/live ratios were observed in adhered *S. epidermidis* cells on the structured ME wing (*p* = 0.4172) and PEG replica samples (*p* = 0.9770), when compared with cells adhered on non-structured PTFE control surfaces.

When assessing the level of antibacterial activity of the ME wing compared to the PEG replica, it was found that the latter possessed the same ratio of dead/live cells as the original ME wing. No significant differences (*p* = 0.9801) were observed in cell dead/live ratios of *P. fluorescens* following 30 min interaction with ME wing and PEG replica supports. As with the ME wing tested here, no significant cell death was observed against Gram-positive *S. epidermidis* when subjected to PEG replicas of the wing. As mentioned, this was thought to be due to differences in their cell membrane characteristics compared to Gram-negative species [19,67]. The bacterial cell surface coverage was compared for the ME wing and the PEG replica, for both *P. fluorescens* and *S. epidermidis* (Supplementary Materials Figure S11). There was lower surface coverage for both test organisms on the PEG samples (9.2% and 3.5 % for *P. fluorescens* and *S. epidermidis*) compared to the wing (26.3% and 17.5% for *P. fluorescens* and *S. epidermidis*). However, despite the lower coverage, the D/L ratios of cells were similar for both test species on the ME wing and its PEG replica. Supplementary Materials Figure S12 shows representative micrographs of the dead/live cell staining analysis and Supplementary Materials Figure S13 shows SEM images of *P. fluorescens* on an ME wing and the corresponding secondary PEG mold. This observation provides further evidence that the replicated microstructured surface, as described in this study, was not only structurally comparable to the ME wing specimen, but also capable of inducing cell death in Gram-negative cells. The compositional uniformity of the PEG replica, unlike the complex protein-like composition attributes of an ME wing, also suggests that the cell death attributed to natural or synthetic microstructure could be inherently a result of the mechanical interaction of the cell with the pillars and not from a potential combined biochemical process.

## 4. Conclusions

The replication of micron-scale pillar arrays from the five cicada wing samples was achieved using UV-curable PEG. Using PEG as both the primary and secondary mold permitted the replication of the samples with ≤10 pillars/µm^2^. The increased density and surface-to-volume ratio of the ME and AY arrays resulted in increased interfacial adhesion between the primary and secondary molds, making mold separation more challenging. Interfacial adhesion was sufficiently lowered with the use of PPG, as a primary mold allowed PEG secondary molds to be removed without feature loss. The reduced surface energy of the PPG primary mold, in addition to its higher chain length and lower Young’s modulus, contributed to successful demolding. A crucial element of the success of this work was the design and use of the replica molding chamber. By preventing the spreading of the PPGDA prepolymer off the surface of the wing samples, successful replication was achieved. The chamber has broadened the scope of materials which can be used to mold cicada wings. The full potential for the chamber to aid the replication of microstructured surfaces must be further investigated. Finally, we were able to demonstrate that the antibacterial effect of the PEG replica is comparable to the ME wing, at least against the Gram-negative bacterial species used for this work.

## Figures and Tables

**Table 1:** Feature sizes and water contact angles (WCAs) for the wings, primary molds, and secondary molds.

Species	ME ^	AY	TO	BTR	YTR
Wing sample					
Diameter (nm)	148 ± 9	209 ± 20	314 ± 43	817 ± 105	854 ± 104
Height (nm)	215 ± 19	191 ± 22	517 ± 85	2163 ± 402	2368 ± 473
Aspect Ratio	~1:1.45	~1:0.91	~1:1.64	~1:2.64	~1:2.77
WCA (^o^)	117 ± 4	103 ± 9	108 ± 4	113 ± 4	110 ± 2
Primary mold					
Diameter (nm)	131 ± 9	156 ± 14	273 ± 32	720 ± 82	720 ± 148
Pore depth (nm)	184	173	447	2055	2282
WCA (^o^)	39 ± 9	73 ± 6	58 ± 14	53 ± 7	45 ± 2
Secondary mold					
Diameter (nm)	142 ± 7	156 ± 10	286 ± 34	897 ± 115	742 ± 97
Height (nm)	193 ± 19	NA ^+^	471 ± 81	2146 ± 501	2070 ± 414
Aspect Ratio	~1:1.35	NA ^+^	~1:1.65	~1:2.39	~1:2.79
WCA (^o^)	41 ± 3	36 ± 1	NA *	51 ± 6	71 ± 2

^ Molded from a PPG primary mold in the molding chamber; ^+^ Tilt image unable to be obtained to determine accurate height measurements; * WCA could not be calculated.

## Data Availability

Raw data available from corresponding author upon request.

## Supplementary Materials

The following are available online at https://www.mdpi.com/article/10.3390/ma14081910/s1, Figure S1 —AutoCAD diagram of stainless-steel molding chamber used for replica molding of ME wing, planar PPGDA, and planar PEGDA samples. Figure S2—Tilted SEM images of the cicada wings (all scale bars are 1 μm) ME, AY, TO, BTR, YTR from top to bottom (images tilted at 45°). Figure S3—SEM top-down images of primary molds of ME (PEG and PPG), AY, TO, BTR and YTR (all scale bars are 1 μm). Table S1—Contact angle measurements and calculated surface free energy on planar PPG and PEG molds using the OWRK model. Figure S4 - Snap cleave SEM images of the primary molds from the different wing samples. Figure S5—Photos demonstrating the drop cast method of molding of the BTR wing (left) to form the primary mold. The fixed primary mold (right) being used to fabricate the PEG wing replica. Figure S6—Images of contact angles for surface free energy measurements of PEG and PPG using water and diiodomethane as test liquids. Figure S7—Image of secondary PEG molds produced from the two different methods described in the paper. Figure S8—SEM images showing larger micron scale features present on the cicada wing replicas. Figure S9—3D models of the cross section of the arrays on the wings created using OnShape software (top row) and top-down views of the 3D models (bottom row). Figure S10—Graphs showing total surface area, surface area to volume ratio, pillar density per µm^2^ and average spacing at the base of the pillars. Figure S11—Graph showing *P. fluorescens* and *S. epidermidis* surface coverage. Figure S12—Epi-fluorescence microscopy images of *P. fluorescens* and *S. epidermidis* bacteria on surfaces. Figure S13—SEM images of *P. fluorescens* cells on ME wing and PEG replica.

## References

[1] Liu M., Zheng Y., Zhai J., Jiang L. (2010). Bioinspired Super-antiwetting Interfaces with Special Liquid—Solid Adhesion. Acc. Chem. Res..

[2] Hasan J., Roy A., Chatterjee K., Yarlagadda P.K.D. (2019). V Mimicking Insect Wings: The Roadmap to Bioinspiration. ACS Biomater. Sci. Eng..

[3] Jaggessar A., Shahali H., Mathew A., Yarlagadda P.K.D. (2017). Bio-mimicking nano and micro-structured surface fabrication for antibacterial properties in medical implants. J. Nanobiotechnol..

[4] Kelleher S.M., Habimana O., Lawler J., Casey E., Habimana O., Lawler J., O’reilly B., Daniels S., Cowley A. (2015). Cicada Wing Surface Topography: An Investigation into the Bactericidal Properties of Nanostructural Features Cicada wing surface topography: An investigation into the bactericidal properties of nanostructural features. ACS Appl. Mater. Interfaces.

[5] Sun M., Watson G.S., Zheng Y., Watson J.A., Liang A. (2009). Wetting properties on nanostructured surfaces of cicada wings. J. Exp. Biol..

[6] Ivanova E.P., Hasan J., Webb H.K., Truong V.K., Watson G.S., Watson J.A., Baulin V.A., Pogodin S., Wang J.Y., Tobin M.J. (2012). Natural bactericidal surfaces: Mechanical rupture of pseudomonas aeruginosa cells by cicada wings. Small.

[7] Hasan J., Webb H.K., Truong V.K., Pogodin S., Baulin V.A., Watson G.S., Watson J.A., Crawford R.J., Ivanova E.P. (2013). Selective bactericidal activity of nanopatterned superhydrophobic cicada Psaltoda claripennis wing surfaces. Appl. Microbiol. Biotechnol..

[8] Nowlin K., Lajeunesse D.R. (2017). Fabrication of hierarchical biomimetic polymeric nanostructured surfaces. Mol. Syst. Des. Eng..

[9] Bandara C.D., Singh S., Afara I.O., Wolff A., Tesfamichael T., Ostrikov K., Oloyede A. (2017). Bactericidal Effects of Natural Nanotopography of Dragonfly Wing on Escherichia coli. ACS Appl. Mater. Interfaces.

[10] Shahali H., Hasan J., Mathews A., Wang H., Yan C., Tesfamichael T., Yarlagadda P.K.D.V. (2019). Multi-biofunctional properties of three species of cicada wings and biomimetic fabrication of nanopatterned titanium pillars. J. Mater. Chem. B.

[11] Bandara C.D., Ballerin G., Leppänen M., Tesfamichael T., Ostrikov K.K., Whitchurch C.B. (2020). Resolving Bio−Nano Interactions of E. coli Bacteria−Dragonfly Wing Interface with Helium Ion and 3D-Structured Illumination Microscopy to Understand Bacterial Death on Nanotopography. ACS Biomater. Sci. Eng..

[12] Lu N., Zhang W., Weng Y., Chen X., Cheng Y., Zhou P. (2016). Fabrication of PDMS surfaces with micro patterns and the effect of pattern sizes on bacteria adhesion. Food Control.

[13] Vasudevan R., Kennedy A.J., Merritt M., Crocker F.H., Baney R.H. (2014). Microscale patterned surfaces reduce bacterial fouling-microscopic and theoretical analysis. Colloids Surfaces B Biointerfaces.

[14] Spengler C., Nolle F., Mischo J., Faidt T., Grandthyll S., Thewes N., Koch M., Müller F., Bischoff M., Klatt M.A. (2019). Strength of bacterial adhesion on nanostructured surfaces quantified by substrate morphometry. Nanoscale.

[15] Tu Q., Wang J.C., Liu R., Zhang Y., Xu J., Liu J., Yuan M.S., Liu W., Wang J. (2013). Synthesis of polyethylene glycol- and sulfobetaine-conjugated zwitterionic poly(l-lactide) and assay of its antifouling properties. Colloids Surfaces B Biointerfaces.

[16] Perni S., Prokopovich P. (2013). Micropatterning with conical features can control bacterial adhesion on silicone †. Soft Matter.

[17] Xue F., Liu J., Guo L., Zhang L., Li Q. (2015). Theoretical study on the bactericidal nature of nanopatterned surfaces. J. Theor. Biol..

[18] Pogodin S., Hasan J., Baulin V.A., Webb H.K., Truong V.K., Phong Nguyen T.H., Boshkovikj V., Fluke C.J., Watson G.S., Watson J.A. (2013). Biophysical model of bacterial cell interactions with nanopatterned cicada wing surfaces. Biophys. J..

[19] Heckmann T.S., Schiffman J.D. (2020). Spatially Organized Nanopillar Arrays Dissimilarly Affect the Antifouling and Antibacterial Activities of Escherichia coli and Staphylococcus aureus. ACS Appl. Mater. Interfaces.

[20] Román-Kustas J., Hoffman J.B., Reed J.H., Gonsalves A.E., Oh J., Li L., Hong S., Jo K.D., Dana C.E., Miljkovic N. (2020). Molecular and Topographical Organization: Influence on Cicada Wing Wettability and Bactericidal Properties. Adv. Mater. Interfaces.

[21] Román-Kustas J., Hoffman J.B., Alonso D., Reed J.H., Gonsalves A.E., Oh J., Hong S., Jo K.D., Dana C.E., Alleyne M. (2020). Analysis of cicada wing surface constituents by comprehensive multidimensional gas chromatography for species differentiation. Microchem. J..

[22] Bhadra C.M., Truong V.K., Pham V.T.H., Al Kobaisi M., Seniutinas G., Wang J.Y., Juodkazis S., Crawford R.J., Ivanova E.P. (2015). Antibacterial titanium nano-patterned arrays inspired by dragonfly wings. Sci. Rep..

[23] Hazell G., May P.W., Taylor P., Nobbs A.H., Welch C.C., Su B. (2018). Studies of black silicon and black diamond as materials for antibacterial surfaces. Biomater. Sci..

[24] Yamada M., MinouraI K., Mizoguchi T., Nakamatsu K., Taguchi T., Kameda T., Sekiguchi M., Suzutani T., Konno S. (2018). Antibacterial effects of nano-imprinted motheye film in practical settings. PLoS ONE.

[25] Watson G.S., Green D.W., Schwarzkopf L., Li X., Cribb B.W., Myhra S., Watson J.A. (2015). A gecko skin micro/nano structure—A low adhesion, superhydrophobic, anti-wetting, self-cleaning, biocompatible, antibacterial surface. Acta Biomater..

[26] Gao H., Liu Z., Zhang J., Zhang G., Xie G. (2007). Precise replication of antireflective nanostructures from biotemplates. Appl. Phys. Lett..

[27] Ito S., Kasuya M., Kawasaki K., Washiya R., Shimazaki Y., Miyauchi A., Kurihara K., Nakagawa M. (2018). Selection of Diacrylate Monomers for Sub-15 nm Ultraviolet Nanoimprinting by Resonance Shear Measurement. Langmuir.

[28] Ho D., Zou J., Zdyrko B., Iyer K.S., Luzinov I. (2015). Capillary force lithography: The versatility of this facile approach in developing nanoscale applications. Nanoscale.

[29] Kafka J., Matschuk M., Larsen N.B. (2013). Injection molding of high aspect ratio sub-100 nm nanostructures. J. Micromech. Microeng..

[30] Maghsoudi K., Jafari R., Momen G., Farzaneh M. (2017). Micro-nanostructured polymer surfaces using injection molding: A review. Mater. Today Commun..

[31] Huang J., Wang X., Wang Z.L. (2006). Controlled Replication of Butterfly Wings for Achieving Tunable Photonic Properties. Nano Lett..

[32] Seol M.L., Woo J.H., Lee D.I., Im H., Hur J., Choi Y.K. (2014). Nature-replicated nano-in-micro structures for triboelectric energy harvesting. Small.

[33] Zada I., Zhang W., Zheng W., Zhu Y., Zhang Z., Zhang J., Imtiaz M., Abbas W., Zhang D. (2017). The highly efficient photocatalytic and light harvesting property of Ag-TiO_2_ with negative nano-holes structure inspired from cicada wings. Sci. Rep..

[34] Weng C., Wang F., Zhou M., Yang D., Jiang B. (2018). Fabrication of hierarchical polymer surfaces with superhydrophobicity by injection molding from nature and function-oriented design. Appl. Surf. Sci..

[35] Weng C., Yang J., Wang F., Ding T., Zhai Z. (2020). Thermodynamic analysis and injection molding of hierarchical superhydrophobic polypropylene surfaces. J. Polym. Eng..

[36] Yang X., Xiao S., Hu W., Hwu J., Van De Veerdonk R., Wago K., Lee K., Kuo D. (2014). Integration of nanoimprint lithography with block copolymer directed self-assembly for fabrication of a sub-20 nm template for bit-patterned media. Nanotechnology.

[37] Kim J.K., Cho H.S., Jung H.S., Lim K., Kim K.B., Choi D.G., Jeong J.H., Suh K.Y. (2012). Effect of surface tension and coefficient of thermal expansion in 30nm scale nanoimprinting with two flexible polymer molds. Nanotechnology.

[38] Krauss P.R., Chou S.Y. (1997). Sub-10 nm imprint lithography and applications. J. Vac. Sci. Technol. B Microelectron. Nanom. Struct. Process. Meas. Phenom..

[39] Zhang G., Zhang J., Xie G., Liu Z., Shao H. (2006). Cicada wings: A stamp from nature for nanoimprint lithography. Small.

[40] Hong S.-H., Hwang J., Lee H. (2009). Replication of cicada wing’s nano-patterns by hot embossing and UV nanoimprinting. Nanotechnology.

[41] Xie H., Huang H., Peng Y. (2017). Rapid fabrication of bio-inspired nanostructure with hydrophobicity and antireflectivity on polystyrene surface replicating from cicada wings †. Nanoscale.

[42] Kumar G., Tang H.X., Schroers J. (2009). Nanomoulding with amorphous metals. Nature.

[43] Ito S., Kikuchi E., Watanabe M., Sugiyama Y., Kanamori Y., Nakagawa M. (2017). Silica imprint templates with concave patterns from single-digit nanometers fabricated by electron beam lithography involving argon ion beam milling. Jpn. J. Appl. Phys..

[44] Dickson M.N., Liang E.I., Rodriguez L.A., Vollereaux N., Yee A.F. (2015). Nanopatterned polymer surfaces with bactericidal properties. Biointerphases.

[45] Carbaugh D.J., Wright J.T., Rajan P., Kaya S., Rahman F. (2016). Dry photolithography through ultraviolet radiation-induced photo-etching of polymethyl methacrylate. Thin Solid Films.

[46] Chandra D., Taylor J.A., Yang S. (2008). Replica molding of high-aspect-ratio (sub-)micron hydrogel pillar arrays and their stability in air and solvents. Soft Matter.

[47] Suh K.Y., Seong J., Khademhosseini A., Laibinis P.E., Langer R. (2004). A simple soft lithographic route to fabrication of poly(ethylene glycol) microstructures for protein and cell patterning. Biomaterials.

[48] Guo L.J. (2007). Nanoimprint lithography: Methods and material requirements. Adv. Mater..

[49] Copic D., Park S.J., Tawfick S., De Volder M.F.L., Hart A.J. (2011). Fabrication of high-aspect-ratio polymer microstructures and hierarchical textures using carbon nanotube composite master molds. Lab Chip.

[50] Liu X., Wu W., Wang X., Luo Z., Liang Y., Zhou F. (2009). A replication strategy for complex micro/nanostructures with superhydrophobicity and superoleophobicity and high contrast adhesion. Soft Matter.

[51] Gordan O.D., Persson B.N.J., Cesa C.M., Mayer D., Hoffmann B., Dieluweit S., Merkel R. (2008). On Pattern Transfer in Replica Molding. Langmuir.

[52] Hahn M.S., Taite L.J., Moon J.J., Rowland M.C., Ruffino K.A., West J.L. (2006). Photolithographic patterning of polyethylene glycol hydrogels. Biomaterials.

[53] Choi S.J., Yoo P.J., Baek S.J., Kim T.W., Lee H.H. (2004). An ultraviolet-curable mold for Sub-100-nm lithography. J. Am. Chem. Soc..

[54] Kim E., Xia Y., Whitesides G.M. (1995). Polymer microstructures formed by moulding in capillaries. Nature.

[55] Rogers J.A., Lee H.H. (2009). Unconventional Nanopatterning Techniques and Applications.

[56] Shahsavan H., Arunbabu D., Zhao B. (2012). Biomimetic modification of polymeric surfaces: A promising pathway for tuning of wetting and adhesion. Macromol. Mater. Eng..

[57] Shahsavan H., Zhao B. (2011). Conformal adhesion enhancement on biomimetic microstructured surfaces. Langmuir.

[58] Amirsadeghi A., Lee J.J., Park S. (2011). Surface adhesion and demolding force dependence on resist composition in ultraviolet nanoimprint lithography. Appl. Surf. Sci..

[59] Min H., Zheng N., Fan Z., Jiang Y., Cheng X. (2019). UV-curable nanoimprint resist with liquid volume-expanding monomers. Microelectron. Eng..

[60] Delaney C., Geoghegan N., Ibrahim H., O’Loughlin M., Rodriguez B.J., Florea L., Kelleher S.M. (2020). Direct Laser Writing to Generate Molds for Polymer Nanopillar Replication. ACS Appl. Polym. Mater..

[61] Wu S. (1982). Polymer Interface and Adhesion.

[62] Park J., Shim G., Lee J., Jang S., Kim H., Choi J. (2018). Evaluation of UV Curing Properties of Mixture Systems with Differently Sized Monomers. Materials.

[63] Sangermano M., Razza N., Crivello J.V. (2014). Cationic UV-curing: Technology and applications. Macromol. Mater. Eng..

[64] Decker C. (2002). Kinetic Study and New Applications of UV radiation Curing. Macromol. Rapid Commun..

[65] Duta L., Popescu A., Zgura I., Preda N., Mihailescu I., Aliofkhazraei M. (2015). Wettability of nanostructured surfaces. Wetting and Wettability.

[66] Wu Y., Zhou S., Wu L. (2016). Fabrication of Robust Hydrophobic and Super-Hydrophobic Polymer Films with Onefold or Dual Inverse Opal Structures. Macromol. Mater. Eng..

[67] Navarre W.W. (1999). Surface Proteins of Gram-Positive Bacteria and Mechanisms of Their Targeting to the Cell Wall Envelope. Microbiol. Mol. Biol. Rev..

