# Supplementary material for: Biomimetic Polymer Surfaces by High Resolution Molding of the Wings of Different Cicadas
Source: Materials (Basel). 2021 Apr 11;14(8):1910. doi: 10.3390/ma14081910 (PMC8068934; doi:10.3390/ma14081910)
Supplement: Supplementary file 1 [file materials-14-01910-s001.zip › materials-1147900-supplementary.pdf]

materials

MDPI

Biomimetic polymer surfaces by high resolution molding of the wings of different cicadas

Graham Reid[1], *James C. McCormack*[1], *Olivier Habimana*[2], *Fabian Bayer*[3], *Catherine Goromonzi*[3], *Eoin Casey*[4], *Aidan Cowley*[3†] *and Susan M. Kelleher* [1,5*]

[1] School of Chemistry, University College Dublin, Dublin 4, Ireland
[2] School of Biological Sciences, The University of Hong Kong, Pokfulam Road, Hong Kong SAR, China
[3] School of Electronic Engineering, Dublin City University, Glasnevin, Dublin 9, Ireland
[4] School of Chemical and Bioprocess Engineering, University College Dublin, Dublin 4, Ireland
[5] School of Chemical Sciences, Dublin City University, Glasnevin, Dublin 9, Ireland

[†] Current address: European Astronaut Centre, Linder Höhe, D-51147 Cologne, Germany

*Corresponding author: susan.kelleher@dcu.ie

Keywords: Replica molding; cicada wings; biomimetic; microstructured surfaces

[†] Current address: European Astronaut Centre, Linder Höhe, D-51147 Cologne, Germany

Supplementary Information

To control the dimensions of the UV-curable polymer molds a chamber was designed. By curing PDMS gaskets with a desired thickness to control the z dimension, the x and y dimensions can simply be cut using a scalpel. Our chamber was designed using AutoCAD software and manufactured by Steger GmbH, Germany. For this work, the ME wing primary and secondary molds were fabricated using the molding chamber described in **Figure S1**.

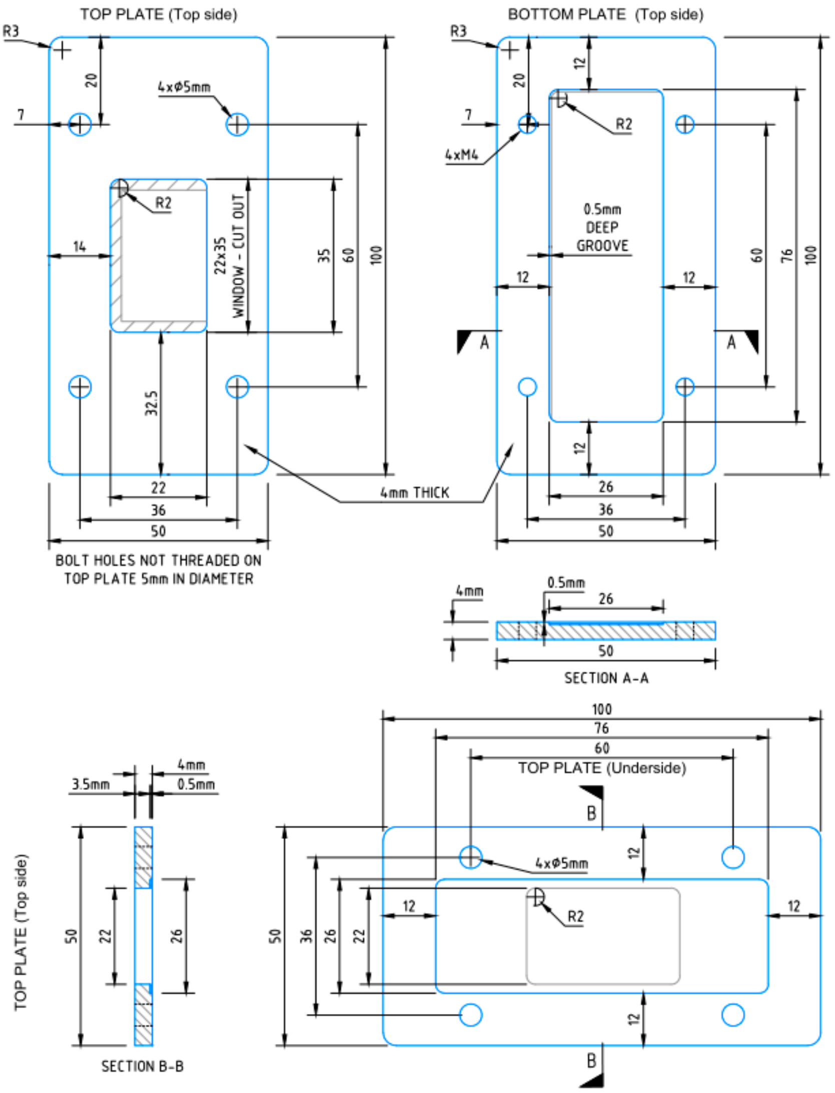

Figure S1. *AutoCAD diagram of stainless-steel molding chamber used for replica molding of ME wing, planer PPGDA, and planer PEGDA samples.*

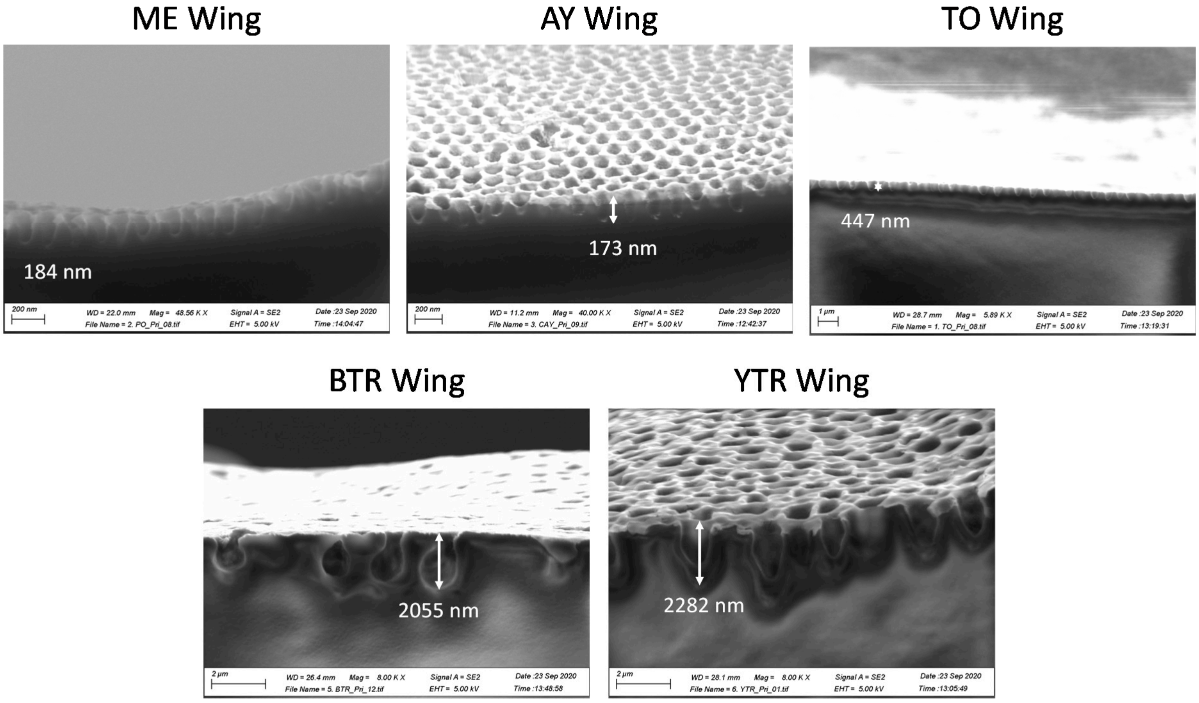

Figure S2. *Snap cleave SEM images of the primary molds from the different wing samples.*

Figure S3 shows images of a fixed wing and primary mold samples used to fabricate the BTR wing topography using PEGDA. By fixing the wing sample to a glass slide, a controlled volume of prepolymer was cast onto the wing using a micropipette. This was then cured using UV-irradiation. The resulting cured PEG negative of the wing was then separated from the template and fixed to a glass slide. The same method was then used to produce a PEG replica of the original wing topography, using the primary PEG mold as the template.

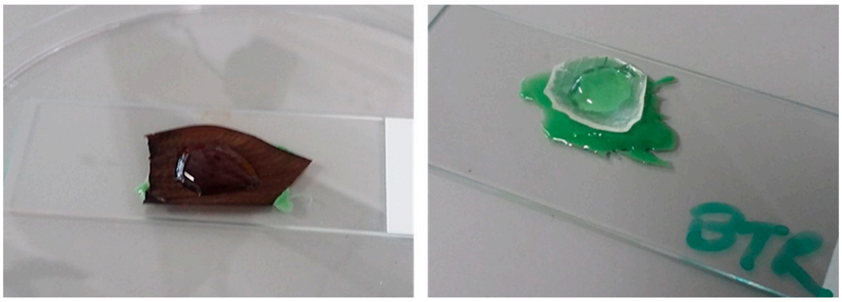

Figure S3. *Photos demonstrating the drop cast method of molding of the BTR wing (left) to form the primary mold. The fixed primary mold (right) being used to fabricate the PEG wing replica.*

Table S1. Contact angle measurements and surface free energy on planar PPG, PEG molds using the OWRK model.

Polymer	Contact Angle (deg)		Surface Free Energy ($mN.m^{-1}$)		
	Water	Diiodomethane	γ^d	γ^p	γ^{total}
**PEG**	46 ± 2	31 ± 2	43.7	18.5	62.2
**PPG**	66 ± 3	45 ± 3	36.8	10.5	47.3

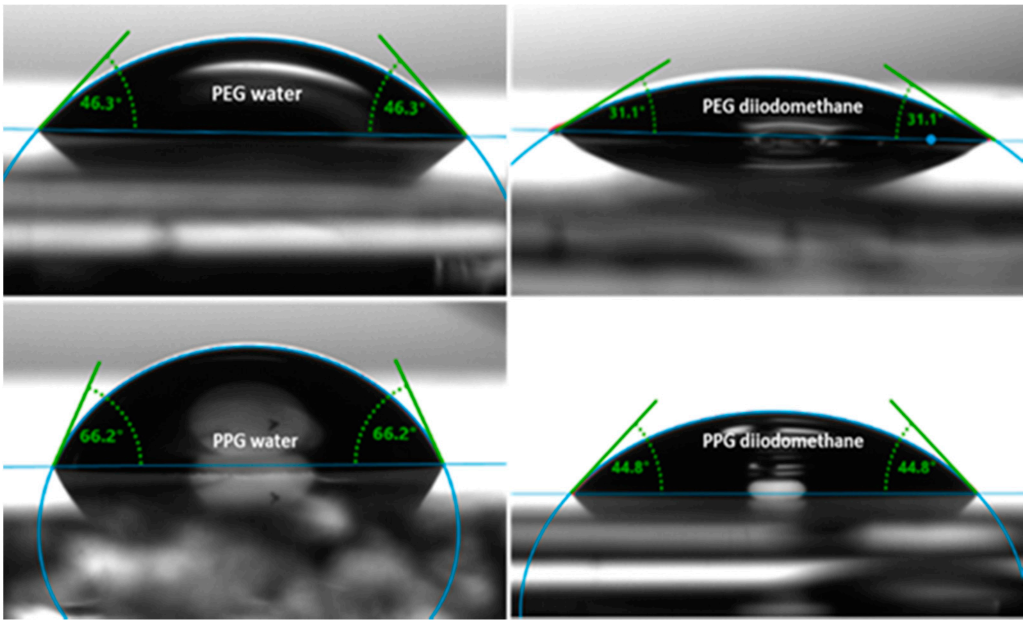

Figure S4. *Images of contact angles for surface free energy measurements of PEG and PPG using water and diiodomethane as test liquids*

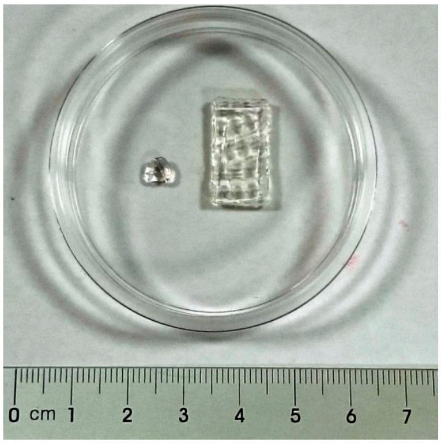

Figure S5. *Image of secondary PEG molds produced from the two different methods described in the paper. Left shows the PEG replica from the drop-cast method, right produced using the molding chamber*

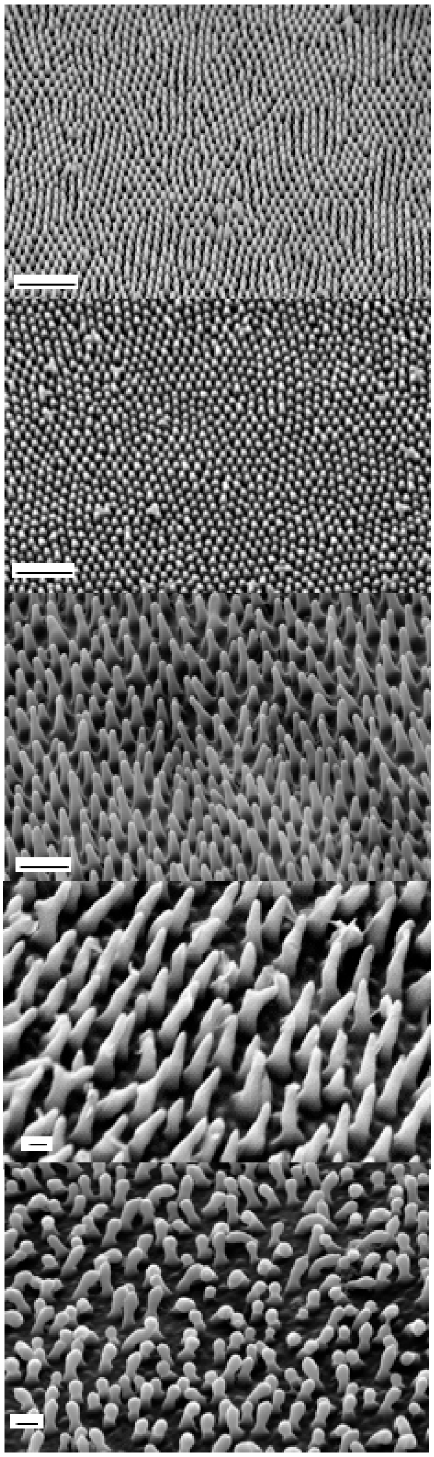

Figure S6. *Tilted SEM images of the cicada wings (all scale bars are 1 μm) ME, AY, TO, BTR, YTR from top to bottom (images tilted at 45°)*

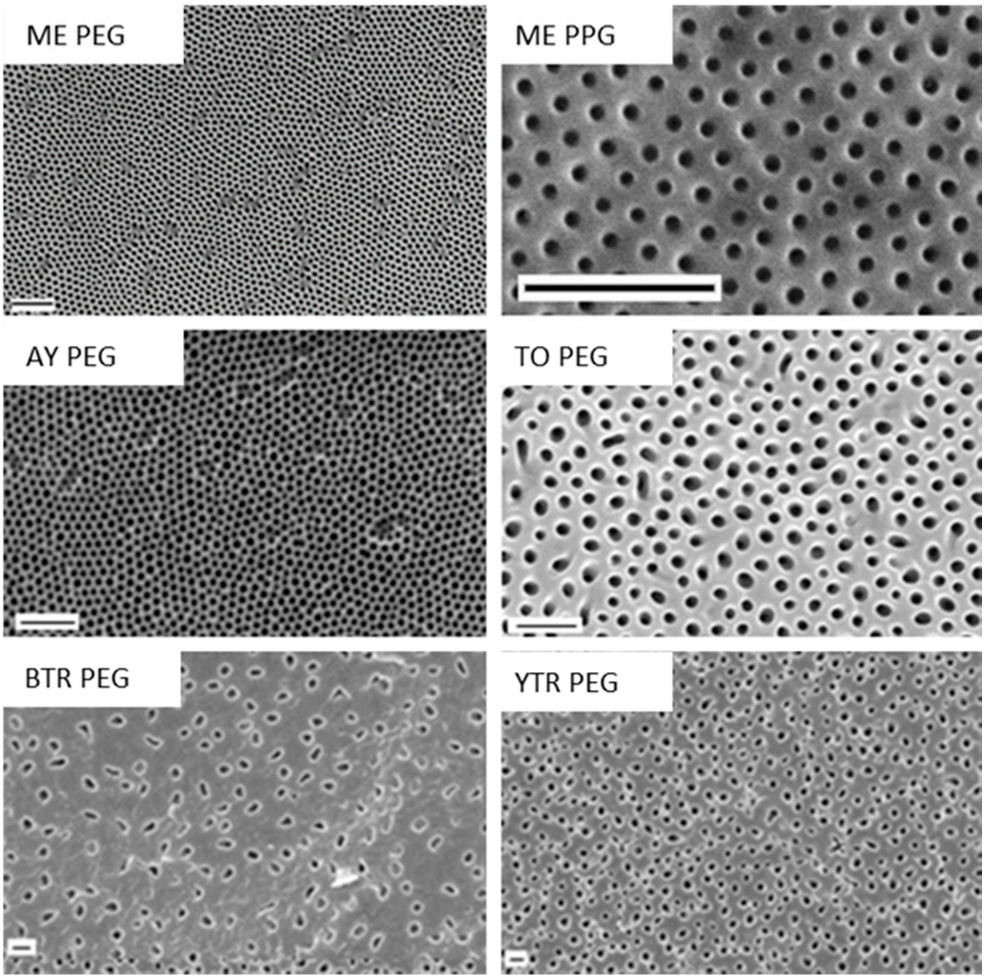

Figure S7. *SEM top-down images of primary molds of ME (PEG and PPG), AY, TO, BTR and YTR (all scale bars are 1 µm)*

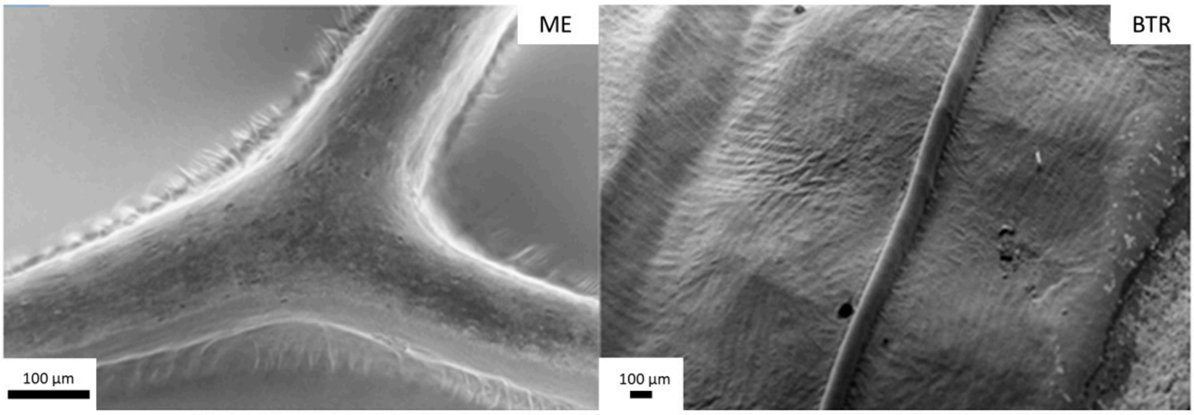

Figure S8. *SEM images showing larger micron scale features present on the cicada wing replicas*

OnShape software was used to create 3D models of the different wing topographies. **Figure S9**, shows the side profiles and top-down models that were used to calculate approximate surface area of mold topographies. This work was carried out to ascertain if there was any connection between the surface area of the wing molds and difficulties encountered when separating PEG-PEG primary-secondary molds. The major differences between the molds were the surface area to volume ratios, density of pillars per μm^2 and spacing between the base of adjacent pillars, as shown in **Figure S10**. The ME and the AY replica molded PEG samples could not be separated without significant damage to primary and secondary molds.

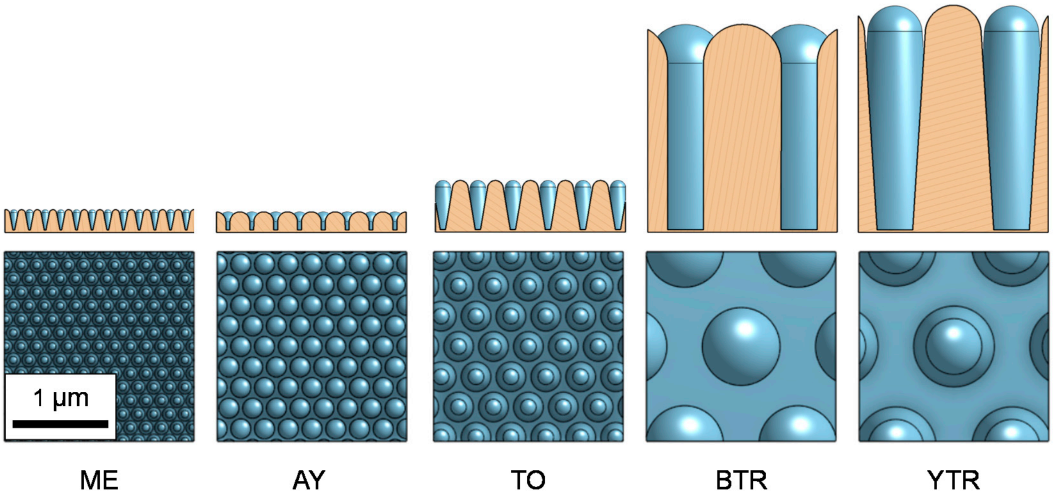

Figure S9. *3D models of the cross sections of the pillar arrays on the wings created using OnShape software (top row) and top-down views of the 3D models (bottom row)*

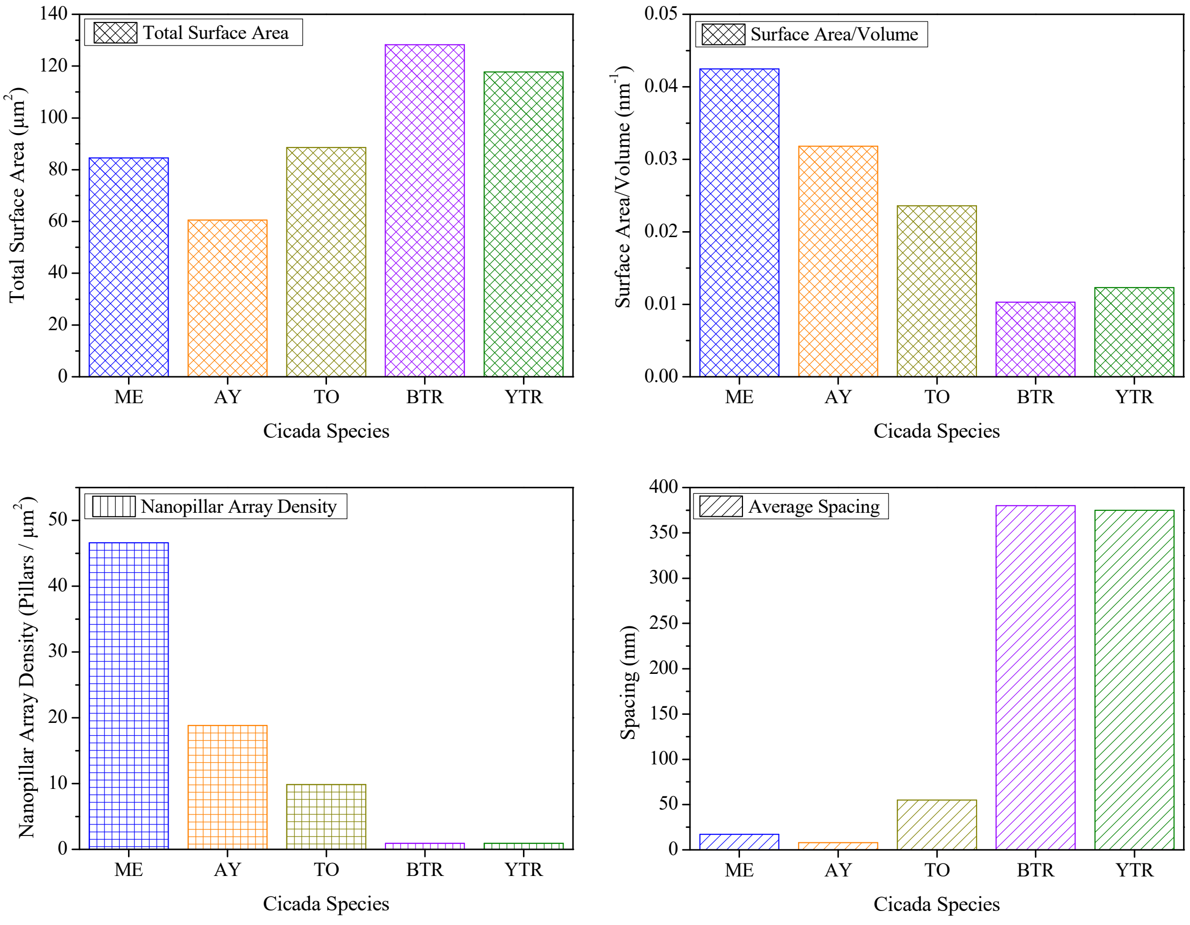

Figure S10. *Graphs displaying the total surface area, surface area to volume ratio, pillar density per μm² and average spacing at the base of the pillars on the wings*

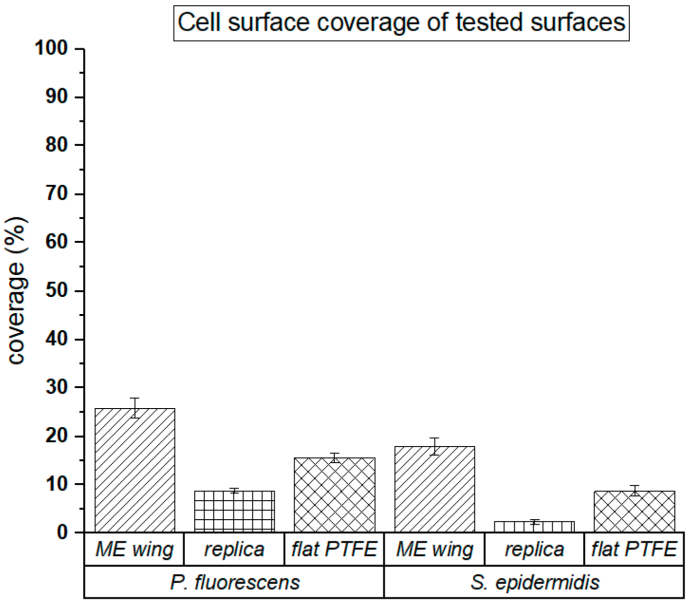

Figure S11. *Graph showing P. fluorescens and S. epidermidis surface coverage of tested surfaces, ME wing, PEG wing replica and flat PTFE control.*

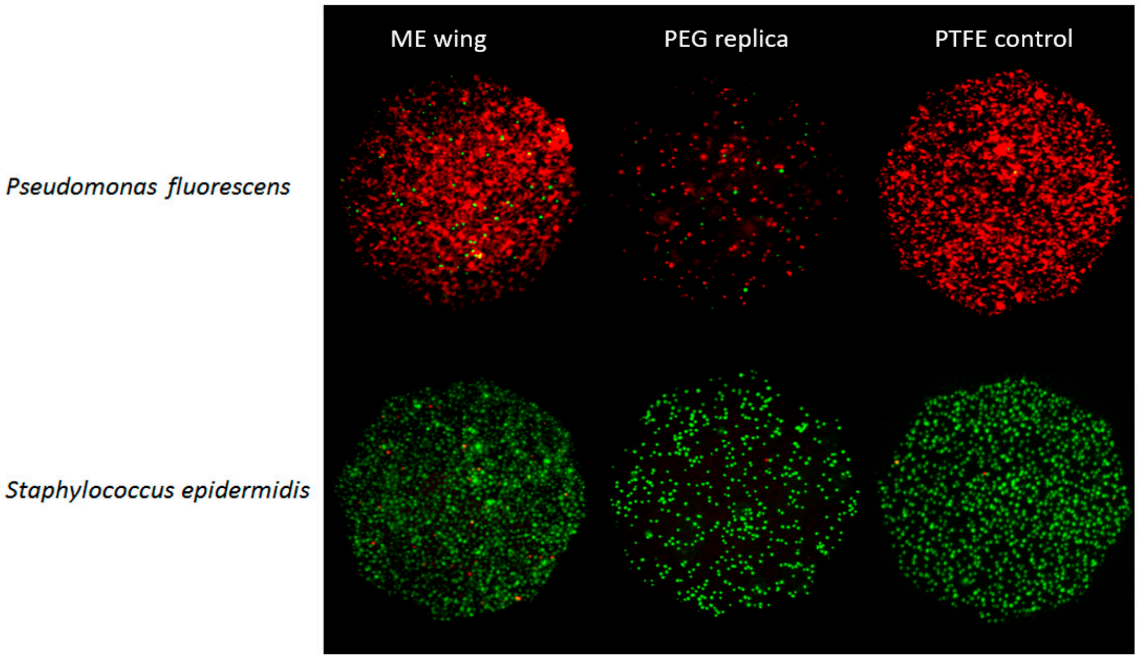

Figure S12. *Epi-fluorescent images of P. fluorescens and S. epidermidis bacteria on surfaces*

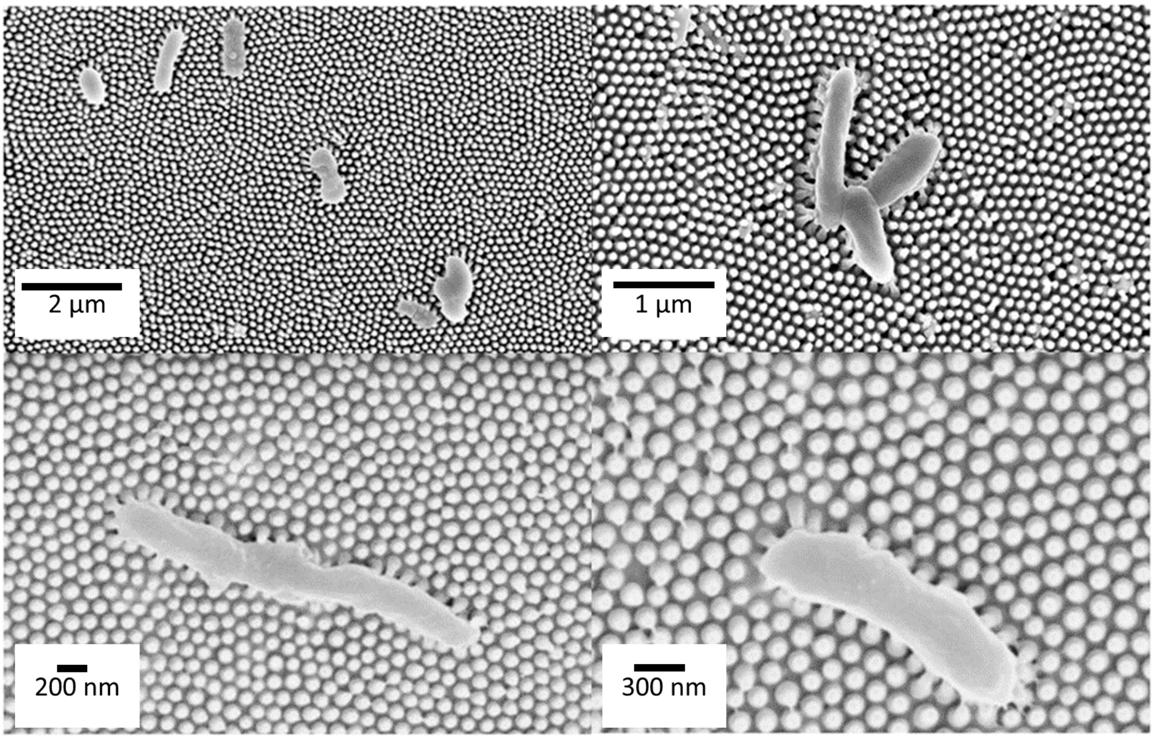

Figure S13. *Top-down SEM images of P. fluorescens bacteria on ME wing sample (top row) and ME replica PEG sample (bottom row).*
